# A three monoclonal antibody combination potently neutralizes multiple botulinum neurotoxin serotype F subtypes

**DOI:** 10.1371/journal.pone.0174187

**Published:** 2017-03-21

**Authors:** Yongfeng Fan, Consuelo Garcia-Rodriguez, Jianlong Lou, Weihua Wen, Fraser Conrad, Wenwu Zhai, Theresa J. Smith, Leonard A. Smith, James D. Marks

**Affiliations:** 1 Department of Anesthesia and Perioperative Care, University of California, San Francisco, Zuckerberg San Francisco General Hospital and Trauma Center, San Francisco, California, United States of America; 2 Molecular and Translational Sciences Division, United States Army Medical Institute of Infectious Diseases (USAMRIID), Fort Detrick, Maryland, United States of America; 3 Medical Countermeasures Technology, United States Army Medical Research Institute of Infectious Diseases, Fort Detrick, Maryland, United States of America; National Cancer Institute, UNITED STATES

## Abstract

Human botulism is primarily caused by botulinum neurotoxin (BoNT) serotypes A, B and E, with around 1% caused by serotype F (BoNT/F). BoNT/F comprises at least seven different subtypes with the amino acid sequence difference between subtypes as high as 36%. The sequence differences present a significant challenge for generating monoclonal antibodies (mAbs) that can bind, detect and neutralize all BoNT/F subtypes. We used repertoire cloning of immune mouse antibody variable (V) regions and yeast display to generate a panel of 33 lead single chain Fv (scFv) mAbs that bound one or more BoNT/F subtypes with a median equilibrium dissociation constant (K_D_) of 4.06 × 10^−9^ M. By diversifying the V-regions of the lead mAbs and selecting for cross reactivity we generated five mAbs that bound each of the seven subtypes. Three scFv binding non-overlapping epitopes were converted to IgG that had K_D_ for the different BoNT/F subtypes ranging from 2.2×10^−8^ M to 1.47×10^−12^ pM. An equimolar combination of the mAbs was able to potently neutralize BoNT/F1, F2, F4 and F7 in the mouse neutralization assay. The mAbs have potential utility as diagnostics capable of recognizing the known BoNT/F subtypes and could be developed as antitoxins to prevent and treat type F botulism.

## Introduction

Botulism occurs in infants and adults and is caused by botulinum neurotoxin (BoNT), the most poisonous substance known. [1,2]. Botulism is characterized by flaccid paralysis, which if not rapidly fatal requires prolonged hospitalization in an intensive care unit and mechanical ventilation. The catalytic domain of BoNT is the light chain (LC), a heavy chain (HC) is comprised of a translocation domain (H_N_) and a receptor-binding domain (H_C_). There are at least seven distinct BoNT serotypes (A-H) [3–5], defined by their neutralization by serotype specific antitoxin; an antitoxin against one serotype will not neutralize another serotype [6,7]. For six serotypes (A-F), there also exist multiple subtypes, which can vary at the amino acid level by a few percent to up to 36% for BoNT/F [8–10]. Subtype sequence differences may result in changes in surface epitopes that can cause a reduction in antitoxin potency [11].

Four BoNT serotypes (A, B, E, and F) cause virtually all human botulism. BoNTs are also classified as Category A biothreat agents, HHS Tier 1 select agents/toxins, one of the seven highest-risk threat agents for bioterrorism [12]. The only treatment for botulism is antitoxin [13]. Equine antitoxin [14,15] and human botulism immune globulin [16,17] are currently licensed to treat adult and infant botulism respectively. Human botulism immune globulin (BabyBIG) is produced by plasmaphersing immunized laboratory personnel who are at risk of exposure to BoNT [18]. Although human botulism immune globulin (licensed for serotypes A and B only) has been shown to be both safe and effective for treating infant botulism scaling of this product to treat adult botulism or for the biothreat drug repository is not feasible [18]. A heptavalent (serotypes A-G) equine botulism antitoxin (HBAT) produced from immunized horses is also licensed in the United States for the treatment of botulism [19]. As a foreign protein, HBAT is immunogenic, and hypersensitivity reactions, including serum sickness and asystole, have been reported [19]. HBAT is an immunoglobulin fragment antigen-binding (Fab’)_2_ whose seven serotype-specific components have short serum half-lives (7.5–34.2 hours), which preclude its effective use for prophylaxis and may predispose to relapse of botulism after treatment [20]. As an alternative, highly potent human monoclonal antibody-(mAb)-based antitoxins composed of three mAbs [21] are being developed for serotypes A, B, C, D and E, with the most advanced (serotype A) having completed Phase 1 human testing [22]. These mAbs bind non-overlapping epitopes on the BoNT molecules of the different BoNT subtypes [23,24].

For this work, we sought to generate a panel of mAbs that bound most, or all, of the highly disparate (up to 36% different at the amino acid level) BoNT/F sub-serotypes for the purpose of generating a therapeutic antitoxin.

## Materials and methods

### Ethics

The USAMRIID Institutional Animal Care and Use Committee approved the animal care and use protocol to conduct the animal studies reported here. Research was conducted under an IACUC approved protocol in compliance with the Animal Welfare Act, PHS Policy, and other Federal statutes and regulations relating to animals and experiments involving animals. The facility where this research was conducted is accredited by the Association for Assessment and Accreditation of Laboratory Animal Care, International (AAALAC/I) and adheres to principles stated in the Guide for the Care and Use of Laboratory Animals, National Research Council, 2011. The specific national regulations and guidelines to which this animal care and use protocol adheres are the following: (1) 7 USC, Sections 2131–2159, Chapter 54 “Animal Welfare Act”, and (CFR, Chapter 1, Subchapter A, Parts 1–4 “Animal Welfare Regulations”; (2) Health Research Extension Act of 1985, Public Law 99–158 “Animals in Research” and the Public Health Service Policy in Humane Care and Use of Laboratory Animals; (3) Biosafety in Microbiological and Biomedical Laboratories, 5th Edition, NIH, Human and Health Services Publication (CDC) 21–112; (4) Army Regulation 40–33 “The Care and Use of Animals in DOD Research, Development, Test and Evaluation or Training Programs” and (5) DOD Instruction 3216.01 “Use of Animals in DOD Programs”. DOD uses the “The Guide for the Care and Use of Laboratory Animals”, 8th Edition, Institute for Laboratory Animal Research, National Research Council, as a guideline for evaluation and accreditation of program and it is based on the actual national regulations and guidelines for animal care and use programs. The animals used in this study were euthanized using carbon dioxide gas following the AVMA Guidelines on Euthanasia prior to spleen removal.

The University of California, San Francisco (UCSF) Institutional Review Board approved the human use protocol used for the studies described here. Human donors were laboratory workers being immunized to work with BoNT who were recruited via an informational letter and who signed informed consent.

### Materials

*Saccharomyces cerevisiae* strain EBY100 was used for library construction and BoNT/F domain display. *Escherichia coli* DH5α was used for subcloning and preparation of plasmid DNA, while the strain BL21 was used for BoNT/F fragment expression. Chinese hamster ovary (CHO) cells were used for immunoglobulin G (IgG) expression. Yeast Peptone dextrose (YPD) medium was used for growth of EBY100 cells. Selective growth dextrose casamino acids media (SD-CAA) was used for selection of pYD4-transformed EBY100 as previously described [25] and selective growth galactose casamino acids media (SG-CAA), for induction of scFv expression on the surface of EBY100. 2 x YT was used for BL21 and DH5α growth. Pure holotoxin BoNT/F1 and polyclonal rabbit anti-BoNT/F antibody were purchased from Metabiologics Inc. (Madison, WI, USA). Clostridial culture supernatants containing unpurified BoNT holotoxins BoNT/Bf2, Af4, F6, and F7 were expressed from strains An436, SU1904, Eklund 202F, and Sullivan, respectively. BoNT/F5 culture supernatant was a kind gift of Susan Maslanka and Brain Raphael at the Centers for Disease Control and Prevention. Mouse anti-SV5 antibody was purified from a hybridoma cell line and labeled with AlexaFluo-488 or AlexaFluo-647 labeling kit (Invitrogen, Carlsbad, CA, USA). All the secondary antibodies including PE (APC)-conjugated goat anti human-Fc, goat anti-mouse Fc and goat anti-human F(ab) were purchased from Jackson ImmunoResearch Laboratories (West Grove, PA).

### Protein expression and purification

The cDNA of 14 BoNT/F fragments including F1-7 N-terminal portion of the light chain (LC-H_N_) and F1-7 C-terminal portion of the heavy chain (H_C_) were amplified from synthetic BoNT/F1-7 genes. All the cDNAs were subcloned into the plasmid pET28b with a hexahistidine (His) tag (for the FLC-H_N_ fragments, F1H_C_ and F7H_C_) or a maltose binding protein (MBP) tag (F2-6H_C_). The fragments were expressed in the *E*. *coli* strain BL21 and purified as described [26]. The MBP-tagged BoNT/F H_C_ fragments were purified using the agarose Dextrin Sepharose High Performance (GE Healthcare, Pittsburg, PA, USA), eluted into PBS with 10mM maltose. The His-tagged fragments were purified with a Ni-NTA agarose (Qiagen, Valencia, CA). The recombinant proteins were dialyzed with PBS buffer overnight to remove imidazole or maltose prior to use. IgG were generated by subcloning V_H_ and V_k_ genes into a mammalian expression vector with human heavy and kappa light chain constant regions, establishment of stable CHO cell lines by transfection and purification of IgG by Protein G chromatography as previously reported [21].

### Mouse immunization and spleen harvest

For the BoNT/F toxin immunization, mice were vaccinated three times at two week intervals (Days 1, 14 and 28) with 10 μg/mouse of catalytically inactive BoNT/ACEF HC. Mice boosted with BoNT/F5 were also vaccinated with BoNT/F5 LC. Mice were boosted twice with 1 μg active toxin (1.0–1.8 x 10^4^ lethal dose 50% (LD_50_) BoNT/F1, BoNT/Bf2, and BoNT/F6; 2.5 x 10^3^ LD_50_ BoNT/Af4; 0.5 x 10^3^ LD_50_ BoNT/F7). BoNT/F5 boosts were done with nontoxic BoNT/F5 LC-H_N_. For BoNT/F LC-H_N_ immunization, 0.25–2.5 μg/mouse of antigen were subcutaneously injected with adjuvant on Days 1,14 and 28, 0.25–2.5 ug/mouse and 0.5–5.0 μg/mouse of antigen was intramuscularly injected with adjuvant; at day 42, proteins were intravenously injected in PBS. Antibody titers after the third vaccination ranged from 0.2–10 x 10^−5^. Mice were euthanized and spleens were removed 3 to 5 days after their final vaccinations and processed to extract mRNA for scFv library construction.

### Yeast-displayed, scFv antibody library construction and sorting

Total RNA was isolated from the blood of healthy human donors immunized with investigational pentavalent BoNT ABCDE toxoid (formalin-inactivated toxins) or from spleens of immunized mice. cDNA synthesis, V_H_ and V_K_ gene repertoires preparation and library construction were completed as previously described [26]. Library size of resultant libraries is given in S1 Table. Finally, the libraries were cultured in SD-CAA media and were induced with SG-CAA media at 18°C over night.

Monoclonal BoNT/F antibodies were isolated as described [25,26] by using 100nM of BoNT/F1 with 1 hour of incubation at room temperature. Then yeasts were incubated with 2 μg/mL of rabbit anti-BoNT/F polyclonal antibody (Metabiologics Inc.) for 60 min. at 4°C, washed, and then incubated with 1 μg/mL of Alexa-488-labeled goat anti-rabbit Fc antibody (Jackson ImmunoResearch Laboratories) and 1 μg/mL Alexa-647-labeled anti-SV5 mAb. After washing, yeasts were sorted by flow cytometry and identified as previously described. [25,26]. For the initial library sorting, positive binding population were gated as many as possible to obtain all the potential binders; but for the libraries for affinity maturation, less than 1% of positive binder were gated to obtain the colonies with improved affinity.

### Measurement of yeast-displayed scFv K_D_

The equilibrium dissociation constant (K_D_) of yeast-displayed scFv was measured by flow cytometry, as previously described [26–29], except that mAbs used were 6F5.1 and 6F11 (for LC-H_N_ binders) or mAbs 6F8 and 6F3 (for H_C_ binders) as secondary antibodies. Briefly, 2x10^6^ yeast were incubated in serially diluted BoNT/F toxin or fragments for 1 hour at room temperature, then with a detection antibody and a R-phycoerythrin (PE)-conjugated goat anti-human or anti-mouse antibody together with Alexa Fluor^®^ 647-labeled SV5 tag antibody. Mean fluorescence intensity (MFI) was measured by flow cytometry as previously described [26,27].

### Ability of mAbs to bind other BoNT/F subtypes

The yeast-displayed scFv were incubated with 100nM BoNT/F1, F2, F3, F5, F6, F7 LC-H_N_ or BoNT/F4 holotoxin (for LC-H_N_ binders) or BoNT/F1, F2, F3, F4, F5, F6, F7 H_C_ individually at room temperature for 1 hour. All subsequent washing and staining steps were performed at 4°C using ice-cold FACS buffer as above. Yeast-displayed scFvs were incubated for 30 minutes with 2 μg/mL of a mixture of Alexa-647 conjugated IgGs 6F5.1 and 6F11 (for LC-H_N_ binders) or mAbs 6F3 and 6F8 (for H_C_ binders) as the secondary antibody for binding detection, together with an Alexa-488 labeled anti-SV5 IgG for detection of scFv expression. Finally, the yeasts were washed using cold FACS buffer and BoNT/F subtype binding was measured by flow cytometry.

### Affinity maturation

The antibodies that potentially bound all seven subtypes of BoNT/F were affinity matured by chain shuffling, followed by error prone PCR mediated random mutagenesis. For the chain shuffling libraries, the V_H_ gene of the scFv was PCR-amplified with primers with gap tails and gel purified. The plasmids containing a pYD4-V_K_ repertoire [25] were linearized by digestion with NcoI/SalI and were purified from an agarose gel by using Geneclean Turbo Kit (MP Biomedicals, Santa Ana, CA, USA). The gap-tailed V_H_ gene was transformed into EBY100 together with the linearized pYD4-V_K_ plasmids using LiAC as described previously [28]. For the random mutagenesis libraries, the cDNA encoding scFvs were PCR-amplified with gap-tailed primers by using Paq5000^™^ DNA polymerase (Agilent, Santa Clara, CA, USA), in which 0.05mM MnCl_2_ was applied to introduce random mutation. The cDNA with mutations was gel purified and transformed into EBY 100 together with NcoI/NotI digested pYD4 plasmid by LiAC method as described previously [28]. The library size was determined by plating serially diluted transformation mixture on SD-CAA plates. The scFv libraries were induced by culturing in SG-CAA media with 10% SD-CAA for at least 24 hours.

In order to isolate the colonies that had improved affinity to all of the BoNT/F subtypes, each library was sorted at least five times; each subsequent round of sorting used a different BoNT/F subtype, with the antigen concentration decreased from 100nM to 100pM from the first to last round of sorting. After the final round of sorting, the collected yeast clones were grown on SD-CAA plates followed by plasmid isolation and DNA sequencing. K_D_ values were measured and the colonies with highest affinity to each BoNT/F subtype were selected.

### Epitope mapping

cDNA encoding each subtype of the BoNT/F1 LC, H_N_ and H_C_ domains were PCR amplified from the seven synthesized BoNT/F genes. Gel purified cDNA fragments were digested with NcoI/NotI and cloned into the plasmid pYD4. The recombinant plasmids were then used to transform EBY100 by LiAC, which were grown on SD-CAA plate for 72 hours. Individual colonies were picked, grown in SD-CAA medium and were induced with SG-CAA medium at 18°C for 48 hours to express the BoNT/F domain on the yeast surface. Yeast colonies were incubated with 2 μg/ml one of the BoNT/F IgGs at room temperature for 1 hour, followed by PE-conjugated goat anti-mouse IgG together with Alexa-488 labeled anti-SV5 IgG at 4°C for 30 minutes. Yeast clones were washed and assayed by flow cytometry.

### BoNT/F modeling and comparison

BoNT/F1 was modeled on BoNT/A crystal structure (pdb ID: 2NZ9) using the software UCSF-Chimera [30]. The modeled LC was replaced by the crystal structure of BoNT/F LC (pdb ID: 2A97), leading to a BoNT/F1 model containing an experimentally determined structure of LC and a modeled F_N_ and F_C_. In order to compare the structure of BoNT/F subtypes, the amino acid sequence of BoNT/F1 was aligned with the other subtypes using the online tool “MultAlin”, (http://multalin.toulouse.inra.fr/multalin), [31]. The alignment was overlaid on the model structure into Esprit 3 online (http://espript.ibcp.fr) [32]. which then. calculated the similarity score based on the physiochemical property of residues. and generated a file with the structure comparison that was edited with Pymol [32]. The difference of the amino acids was coded between red and white; the more different, the more red.

### Mouse neutralization assay

The mouse neutralization assay was performed as described previously [11]. Briefly, mAbs or HBAT were mixed with 1 μg of the indicated BoNT/F subtype and injected into mice. The toxin-exposed mice were observed at least twice daily. Most animals receiving lethal doses of BoNT become moribund within 12 hours, frequently within 4 hours.

## Results and discussion

### Comparison of the surface amino acid differences between BoNT/F subtypes

**Fig 1:**
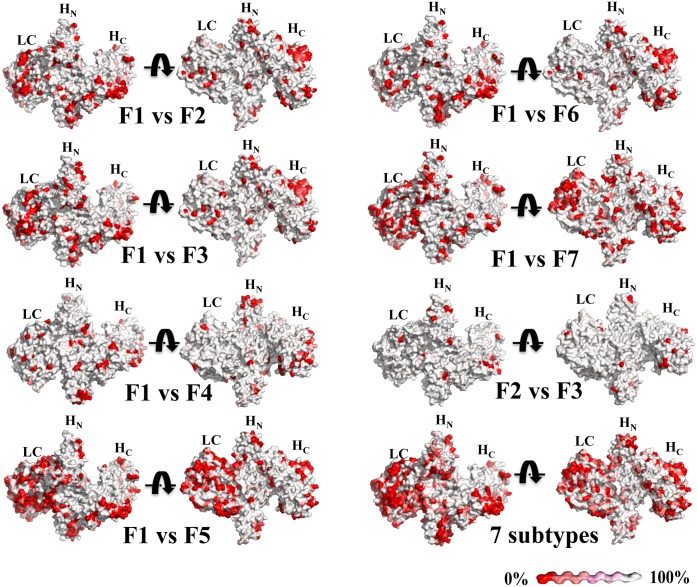
Comparison of the BoNT/F subtypes. The BoNT/F1 model was constructed by merging the BoNT/F1 LC crystal structure (pdb ID: 2A97) and a model of the BoNT/F1 LC-H_N_ model based on the BoNT/A1 crystal structure (pdb ID: 2NZ9). BoNT/F subtypes were compared to the BoNT/F1 by using the tool MultAlin [31] and were visualized using ESPript 3 [32]. The physiochemical similarity of amino acids is indicated on a color scale from white to red. The white indicates 100% identity and the red indicates 0% physiochemical similarity.

The BoNT/F subtypes differ from each other by 1.1% to 36.1% at the amino acid level [10]. BoNT/F1 differs from the other BoNT/F subtypes by 7.7% to 30.1% at the amino acid level. To visualize the impact of these differences on potential mAb binding, we modeled the differences on a model of BoNT/F. The BoNT/F1 model was generated by using a combination of the BoNT/F1 LC crystal structure (pdb ID: 2A97) and a model of the BoNT/F1 HC based on the crystal structure of BoNT/A1 complexed to mAb CR1 (pdb ID: 2NZ9). Differences in the amino acid sequence between BoNT/F1 and the other BoNT/F subtypes results in differences on the BoNT/F surface (Fig 1). These differences are greatest for BoNT/F5 (31.1% different) and BoNT/F7 (26.3% different). The difference between BoNT/F5 and BoNT/F7 are as high as 36.3%. Differences are greatest between the BoNT/F1 LC and the LC of BoNT/F5 (52.4% different) and the LC of BoNT/F7 (36.7% different). In contrast, BoNT/F2 and BoNT/F3 surfaces are highly homologous. Diversity between subtypes makes it challenging to generate high affinity mAbs to all the subtypes.

### Yeast-displayed scFv antibody library

scFv yeast-display libraries were constructed from spleens of immunized mice. Since BoNT/F1 was the only holotoxin commercially available, we produced recombinant BoNT/F domains for immunization of the other subtypes. Synthetic genes encoding the BoNT/F binding domain (H_C_) or translocation domain (H_N_) fused to the light chain (LC) (LC-H_N_) of sub-serotypes F1, F2, F3, F4, F5, F6 and F7 were designed and cloned into plasmid pET28b to contain His tags or MBP tags. His-tagged BoNT/F1 and BoNT/F7 H_C_ and MBP-tagged BoNT/F2, 3, 4, 5, 6 H_C_ were expressed in the cytoplasm and could be purified (S1 Fig). His-tagged BoNT/F1, F2, F3, F5, F6 and F7 LC-H_N_ could be expressed and purified to varying levels of purity (S1 Fig), while BoNT/F4 LC-H_N_ did not express as soluble protein in the cytoplasm. Mice were initially immunized with one of the non-toxic domains and then boosted with either BoNT/F1 holotoxin or the domain used for the initial immunization (S1 Table). BoNT/F2 LC-H_N_ was not used for immunizations due to its high level of identity to BoNT/F1.

Total RNA was isolated from mouse spleens and used to construct scFv libraries in the yeast display vector pYD4 exactly as described [25,26]. Libraries varied in size from 1.6x 10^7^ to 9.0 x 10^7^, with a 3-human donor library mixture of 2.0 x 10^8^ (S1 Table). Total RNA was also isolated from white blood cells from twelve human donors and was used for scFv library construction as previously described [26].

### mAb isolation and initial characterization

After three rounds of sorting, individual colonies were picked and characterized for binding, yielding 33 unique mAbs as determined by DNA sequencing of the scFv genes; 26 of the mAbs were from the murine libraries and seven from the human libraries (Table 1). Four of these mAbs (46A5, 46F8, 47A12, and 54D7) did not bind BoNT/F1 holotoxin. The affinity of the yeast-displayed scFv was measured by flow cytometry, demonstrating a K_D_ to BoNT/F1 ranging from 4.0 × 10^−11^ M to 1 × 10^−7^ M, with a median K_D_ of 4.06 × 10^−9^ M. (Table 1).

**Table 1:** Binding characteristics of selected mAbs against BoNT/F.

mAb name	Origin	Yeast K_D_ (BoNT/F1 Holotoxin)	Domain	Subtype specificity
6F3	Mouse	0.76 × 10^−9^ M	H_C_	F1, F7
6F4	Mouse	2.87 × 10^−9^ M	H_C_	F1
4E17.2	Human	1.50 × 10^−9^ M	H_N_	F1, F2, F3, F4, F5, F6, F7
6F6	Mouse	0.55 × 10^−9^ M	H_N_	F1, F3, F5, F7
6F7	Mouse	1.55 × 10^−9^ M	LC	F1, F3, F4
6F8	Mouse	0.30 × 10^−9^ M	H_C_	F1, F2, F3, F4, F5, F6
6F9	Mouse	0.04 × 10^−9^ M	H_C_	F1, F3, F4,F5
6F10	Mouse	0.12 × 10^−9^ M	LC-H_N_	F1, F6
28H4	Mouse	20.0 × 10^−9^ M	H_C_	F1, F3, F4, F6
30C8	Mouse	0.20 × 10^−9^ M	LC-H_N_	F1, F3, F4, F5
29A2	Mouse	0.99 × 10^−9^ M	LC-H_N_	F1
28C9	Mouse	15.91 × 10^−9^ M	H_C_	F1, F4
32G2	Mouse	5.18 × 10^−9^ M	H_C_	F1, F4
37B4	Human	10.14 × 10^−9^ M	H_C_	F1, F3, F4, F6
37B6	Human	23.75 × 10^−9^ M	H_C_	F1, F2, F3, F4, F5, F6
38B8	Human	18.32 × 10^−9^ M	H_C_	F1, F3, F4, F5, F6
38C1	Human	30.26 × 10^−9^ M	H_C_	F1, F3, F4, F5, F6
38D11	Human	29.13 × 10^−9^ M	H_C_	F1
38F8	Human	29.36 × 10^−9^ M	H_C_	F1, F3, F4, F6
44A12	Mouse	1.09 × 10^−9^ M	H_C_	F1, F4, F7
44C2	Mouse	26.93 × 10^−9^ M	H_C_	F1, F4, F7
44C4	Mouse	29.32 × 10^−9^ M	H_C_	F1, F4, F7
44F4	Mouse	16.85 × 10^−9^ M	H_C_	F1, F4, F7
46.25	Mouse	13.84 × 10^−9^ M	H_N_	F1 F3 F6
46A5	Mouse	No binding	LC-H_N_	F3
46F8	Mouse	No binding	LC-H_N_	F3
46E12	Mouse	4.06 × 10^−9^ M	H_N_	F1 F3 F6 F7
47A12	Mouse	No binding	H_N_	F3, F5, F6
54D7	Mouse	No binding	LC-H_N_	F3, F4, F5, F6, F7
55A9	Mouse	5.25 × 10^−9^ M	H_C_	F1, F4, F7
56E11	Mouse	No binding	LC-H_N_	F3
57A8	Mouse	28.67 × 10^−9^ M	LC-H_N_	F1, F3, F5, F6, F7
57C3	Mouse	>100 × 10^−9^ M	LC-H_N_	F1, F3, F5, F6, F7

BoNT/F subtype cross reactivity of the mAbs was tested by flow cytometry. The scFv 4E17 bound all seven BoNT/F subtypes (Table 1 and S2 Fig). BoNT scFv 6F8 and 37B6 bound six of the seven subtypes but did not show binding to BoNT/F7 (Table 1). scFv 38B8, 38C1, 54D7, 57A8 and 57C3 bound five of the seven subtypes of BoNT/F. The other mAbs showed varying levels of cross reactivity to the other subtypes (Table 1 and S2 Fig).

### Broadening sub-serotype specificity and increasing affinity

**Fig 2:**
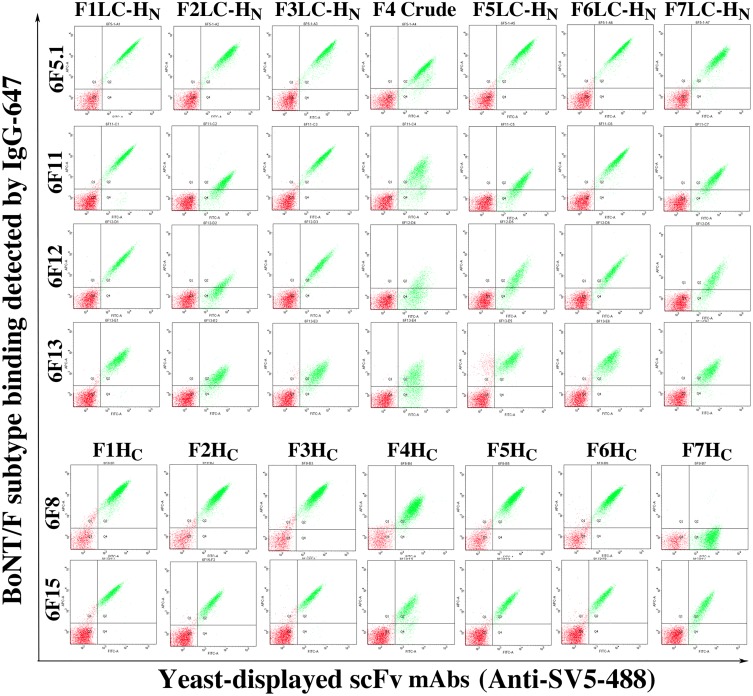
Cross reactivity of the affinity matured mAbs against BoNT/F subtypes. Yeast-displayed single chain Fv were incubated with BoNT/F LC-H_N_ or H_C_ fragments followed by an Alexa-647 labeled secondary IgG for binding detection. An Alexa-488 labeled anti-SV5 IgG was used to detect scFv mAb expression on the yeast surface. For 6F5.1, 6F11, 6F12 and 6F13, BoNT/F LC-HN fragments or BoNT/F4 crude toxin were used (the upper panel) and for 6F8 and 6F15, BoNT/F H_C_ fragments were used.

To broaden subtype specificity and increase affinity, the V-genes of selected scFv were mutated, displayed on yeast and sorted for higher affinity for multiple subtypes [28,29,33,34]. The V_H_ gene of 4E17 was shuffled into a V_L_ library, leading to 4E17.2 (6F5), which showed improved affinity to BoNT/F1 but low affinity to other subtypes. Then V gene of 4E17.2 (6F5) was randomly mutated by error prone PCR and sorted using extremely low BoNT/F1 holotoxin concentrations (100pM), which yielded the scFv 6F5.1 which has a 3.75 to >700 fold increase in affinity for the BoNT/F subtypes with the K_D_ ranging from 0.32 nM for BoNT/F4 to 2.97nM for BoNT/F7 (Table 2, Fig 2). IgG produced from the 6F5.1 scFv showed high affinity binding to all seven BoNT/F subtypes, with K_D_s ranging from 26 pM against BoNT/F4 holotoxin to 204 pM against BoNT/F7 LC-H_N_ (Table 3). In addition, 4E17.2 is a highly cross reactive mAb that also binds H_N_ of BoNT/A, BoNT/B, BoNT/E and BoNT/H [29,33].

**Table 2:** The K_D_ values (x 10^-9^M) of affinity and specificity-matured yeast-displayed scFv for the seven BoNT/F subtypes.

	Antibodies (epitopes)										
Toxin*	4E17.2 (6F5)→6F5.1 (H_N_)		46E12→6F11(H_N_)		46.25→6F1 (H_N_)		47A12→6F13 (H_N_)		6F8 (H_C_)	28H4→6F15(H_C_)	
**F1**	1.5	0.4	23.4	3.81	50.0	6.93	NB	3.63	0.3	12.7	1.77
**F2**	42.3	2.23	NB^#^	93.5	NB	141	NB	97.8	8.8	NB	41.3
**F3**	40.3	2.09	>500	19.8	>500	59.6	>500	13.2	0.44	>500	19.5
**F4**	>250	0.32	NB	3.61	NB	38.9	NB	24.1	5.32	>500	53.4
**F5**	14.1	0.68	NB	>50	NB	49.1	>500	12.8	52.7	NB	196
**F6**	4.88	1.24	>500	8.42	>500	70.3	>500	6.23	4.95	>500	60.3
**F7**	101	2.97	>500	200	NB	>500	>500	39.2	NB	NB	10.5

^#^NB: No binding detected

*For H_N_ binders, BoNT/F1, F2, F3, F5, F6, F7 LC-H_N_ or BoNT/F4 holotoxin were used; For H_C_ binders, BoNT/F1, F2, F3, F4, F5, F6, F7H_C_ were used for K_D_ measurement.

**Table 3:** K_D_ values of selected IgGs to BoNT/F toxin subtypes (x 10^−12^ M) as determined by flow fluorimetry in a KinExA [28].

Toxin	6F5.1	6F11	6F13
BoNT/F1 holotoxin	90.93	1.47	327.31
BoNT/F2 holotoxin	64.22	287.46	22150
BoNT/F3 LC-H_N_	78.76	52.94	2860
BoNT/F4 holotoxin	26.38	85.64	112.17
BoNT/F5 holotoxin	152.01	12850	125.39
BoNT/F6 holotoxin	26.67	142.56	822.63
BoNT/F7 LC-H_N_	204.53	430.60	458.13

A similar strategy was used for scFv 6F8, 37B6, 38B8, 38C1, 54D7, 57A8, 57C3, 46E12, 46.25, 47A12, and 28H4 with the first mutagenesis strategy being light chain shuffling followed by error prone mutagenesis. Selections and sorting were performed by simultaneous incubation of yeast-displayed scFv with two to up to five of the BoNT/F subtype fragments labeled with different fluorescent labels [34]. Using this strategy, the affinity and specificity of scFv 46E12, 46.25, 47A12, and 28H4, were improved to generate scFv 6F11, 6F12, 6F13 and 6F15, respectively. While the starting scFv bound 3–4 subtypes, the resultant scFv bound all seven subtypes (Fig 2), with the K_D_ on yeast ranging from 3.81nM to 200 nM for 6F11, 6.93 nM to >500 nM for 6F12, 3.63 nM to 97.8 nM for 6F13 and 1.77 nM to 60.3 nM for 6F15 (Table 2). Thus for each of these four scFv, it proved possible to impart a measurable K_D_ where previously no detectable binding could be observed (Table 2 and Fig 2 versus S2 Fig). IgG constructed from the engineered 6F11 and 6F13 bound to each of the seven BoNT/F sub-serotypes with K_D_ ranging from 1.47 pM to 12.8 nM for 6F11 and from 112 pM to 32 nM for 6F13 (Table 3). Interestingly, it was not possible to broaden the specificity of scFv 6F8, 37B6, 38B8, 38C1, 54D7, 57A8, or 57C3 to all seven subtypes.

### Epitope determination and overlap analysis

**Fig 3:**
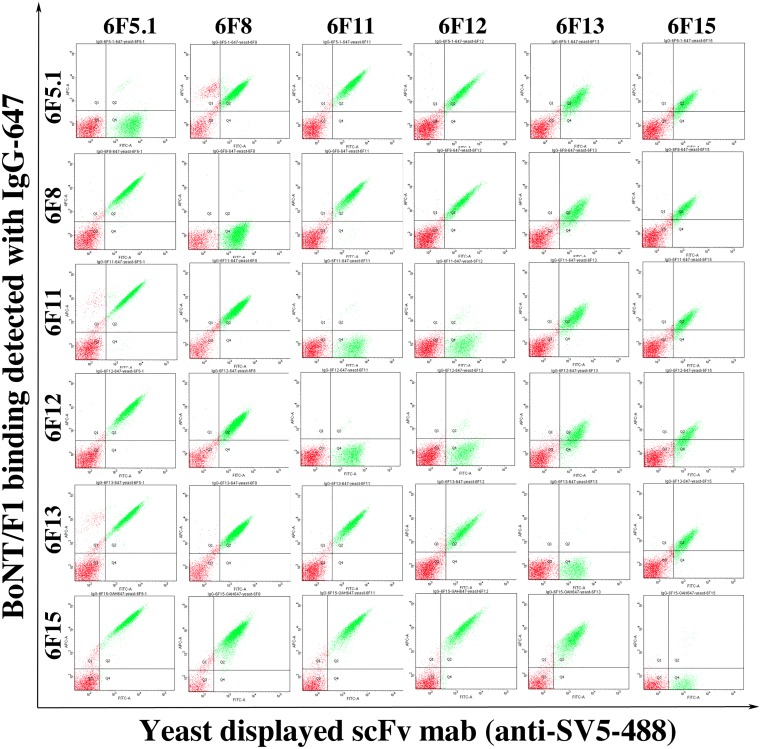
Identification of overlapping and non-overlapping mAb epitopes. Yeast-displayed scFv were incubated with BoNT/F1 holotoxin, followed by incubation with one of six Alexa-647 labeled IgG and Alexa-488 labeled anti-SV5 IgG. mAbs with non-overlapping epitope showed a signal in the Y-axis.

In order to confirm the binding domain of the cross-reactive antibodies, three domains of BoNT/F1, (L_C_, H_N,_ and H_C_) and LC-H_N_ were subcloned into the plasmid pYD4 and displayed on the surface of EBY100. The yeast-displayed domains were incubated with Alexa-647 conjugated IgG and Alexa-488 labeled antiSV5 IgG and detected by flow cytometry. The results demonstrated that 6F5.1, 6F11, 6F12, and 6F13 bound BoNT/F LC-H_N_ and BoNT/F H_N_, indicating that they bound on H_N_ domain, and that 6F8 and 6F15 bound BoNT/F H_C_ (S3 Fig) Only 6F7 bound BoNT/F LC. A sandwich binding assay was designed to determine if the four BoNT/F H_N_ binders shared overlapping or non-overlapping epitopes. BoNT/F1 was captured by a yeast displayed yeast-displayed scFv, and each Alexa labeled IgG was tested for the ability to simultaneously bind the scFv captured BoNT/F1 (Fig 3). 6F11 and 6F12 share an overlapping epitope, while 6F5.1 and 6F13 had epitopes that did not overlap with each other or with the epitopes of 6F11 and 6F12. Similarly, the H_C_ binding 6F8 and 6F15 were able to bind BoNT/F1 simultaneously, indicating that their epitopes on the HC did not overlap (Fig 3).

### Mouse neutralization assay

**Fig 4:**
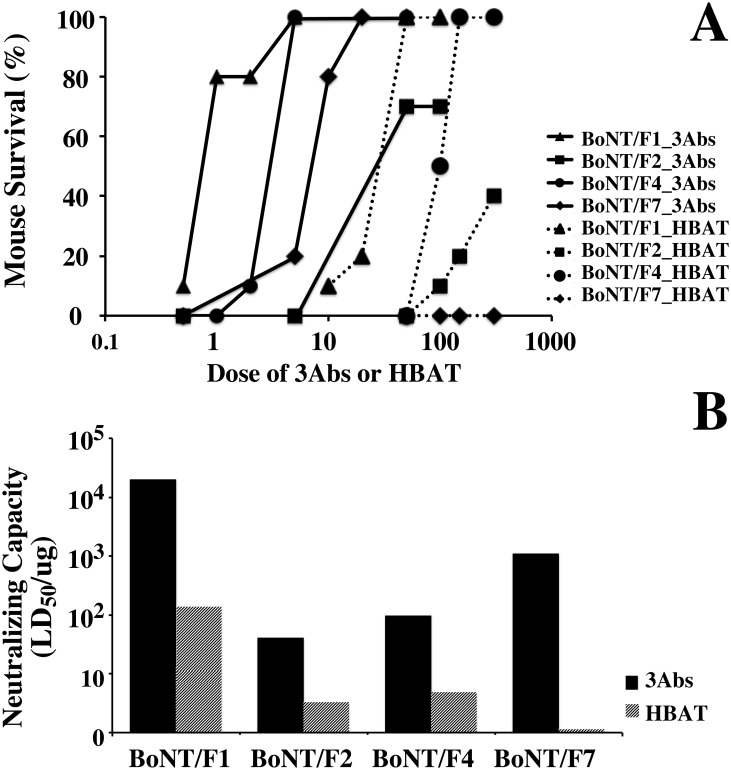
Protection of mice from BoNT/F subtypes challenged by HBAT or triple mAb combination. **(A)** Mouse survival following intoxication with different toxin subtypes. The combination of 6F5.1/6F11/6F13 (3Ab) rescued mice at much lower dosage (the solid lines) than the equine-derived HBAT showing with dash line. **(B)** Neutralizing capacity of HBAT and triple mAb combination (LD_50_/μg), calculating by toxin amount (LD_50_) divided by HBAT or mAbs dosage (μg) that rescue >70% of mice.

Our previous studies demonstrated that potency of *in vivo* BoNT neutralization by mAbs was greatest when three mAbs with high affinity and non-overlapping epitopes were combined [21]. A combination of mAbs 6F5.1, 6F11, and 6F13 were chosen for the mouse neutralization assay due to their ability to bind multiple BoNT/F subtypes, their non-overlapping epitopes, and their relatively high affinity when compared with the other mAbs reported here. Although 6F15 is also cross-reactive to all the seven subtypes, it had lower affinity to BoNT/F4, F5 and F6. HBAT was used as a comparator. Various doses of an equimolar combination of the three mAbs (0.5 μg– 100 μg) or the equine antitoxin (10 μg– 300 μg) were incubated with 1 μg of BoNT/F1, F2, F4, or F7; the mixtures were injected into mice, and the animals were monitored for signs of botulism and mortality. As the antibody:toxin interactions are based on amounts, and not toxicities, of the toxins, an equimolar amount of BoNT/F subtypes (1 μg) was studied rather than an equipotent dose (LD_50_). A broad range of specific activity of the different BoNT/F subtypes (> 40 fold difference in lethality was seen when comparing LD_50_s of 1 μg of BoNT/F4 to 1 μg of BoNT/F1) (Table 4). Five micrograms of the mAb combination completely protected mice that were challenged with 1 μg (20,000 LD_50_) of BoNT/F1 and 1 μg of the mAb combination protected 8/10 mice, indicating a median effective dose (ED_50_) of less than 1 μg (Table 4 and Fig 4). For BoNT/F7, the most frequently reported BoNT/F subtype causing human disease, complete protection of a 1 μg (11,000 LD_50_) challenge dose of BoNT occurred at a mAb dose of 20 μg, with an ED_50_ of less than 10 μg. The mAb combination was least potent for BoNT/F2, with an ED_50_ of approximately 50 μg, likely reflecting the lower affinity of mAb 6F13 for this subtype (22 nM) compared to the other BoNT/F subtypes (112–458 pM, Table 3).

**Table 4:** Neutralization of BoNT/F subtypes in the mouse neutralization assay by HBAT of an equimolar combination of mAbs 6F5.1, 6F11 and 6F13.

	mAbs 6F5.1+6F11+6F13				Equine antitoxin (HBAT)			
BoNT/F subtype	F1	F2	F4	F7	F1	F2	F4	F7
Experimentally-determined LD_50_ of 1 μg of toxin	20000	2000	480	11000	6600^1^	2000	480	11000
Antibody Dose (μg)	Mice surviving/ mice studied							
0.5	1/10	0/10	0/10					
1.0	8/10		0/10					
2.0	8/10		1/10					
5.0	10/10	0/10	10/10	2/10				
10	10/10			8/10	1/10			
20				10/10	2/10			
50		7/10	10/10	9/10	10/10	0/10	0/10	0/10
100		7/10			10/10	1/10	5/10	0/10
150					10/10	2/10	10/10	0/10
300					10/10	4/10		0/10
ED_50_ (ug)^3^	0.5–1.0	20–50	2–5	5–10	20–50	>300^2^	100	>>300^2^

^1^ BoNT/F1 dose reduced as 50% survival not achieved at maximal HBAT dose with 20,000 LD_50_ challenge.

^2^ 300 μg of HBAT protected 9 of 10 mice from 1,000 LD_50_ BoNT/F2 and 344 LD_50_ of BoNT/F7 challenge.

^3^ The ED_50_ is the mAb dose at which greater than 50% of the mice studied survived

The mAb combination was approximately 150 times more potent in neutralizing BoNT/F1 than equine antitoxin on a weight basis and approximately 450 times more potent on a molar basis (IgG molecular weight = 150 kDa; Fab molecular weight = 50 kDa), since 1.0ug of mAbs neutralized 20,000LD_50_ of BoNT/F1. In contrast, 50ug of HBAT was needed to neutralize 6600 LD_50_ of BoNT/F1. This difference would be expected given that only a fraction of polyclonal antitoxins bind the BoNT F1. For BoNT/F2, F4 and F7, the mAb combination also demonstrated much potent neutralizing capacity than HBAT, with the ED_50_ of <50ug for BoNT/F2, < 5.0ug for BoNT/F4 and < 10ug for BoNT/F7. By comparison, the range of ED_50_s of the equine antitoxin for the four BoNT subtypes were more than 100 fold lower than the mAb combination; the lowest ED_50_ was for BoNT/F7, with 300ug of HBAT rescuing 9 of 10 mice with 344 LD_50_ of BoNT/F7 challenge but without showing any protection if mice exposed to 11000 LD_50_ of BoNT/F7 (Table 4 and Fig 4).

## Conclusions

Using repertoire cloning, a panel of murine scFv antibody fragments were generated that bound one or more of the BoNT/F subtypes. Only one of these lead scFv bound all seven BoNT/F subtypes, reflective of the fact that the subtypes differ by up to 36% at the amino acid level [10]. Using yeast display and the simultaneous staining and sorting of yeast-displayed scFv for binding to multiple subtypes, we significantly increased subtype cross-reactivity and affinity. As a result, four mAbs were generated that bound all seven BoNT/F subtypes with high affinity. Interestingly, three of the most cross reactive mAbs, 6F11, 6F12 and 6F13, were those that initially only showed measurable binding to three to four of the BoNT/F subtypes. Broadening specificity occurs through a combination of removing steric clashes, increasing complementarity determining region (CDR) flexibility and generating new contacts such as hydrogen bonds and van der Waals interactions while not changing key contact residues [34,35]. For whatever reason, in the examples here that only proved possible with the less cross-reactive lead mAbs.

The mAbs reported here have potential diagnostic and therapeutic utility due to their subtype cross reactivity. The ability to bind all seven BoNT/F subtypes allows sensitive detection of all known BoNT/F’s and increases the probability that yet-to-be discovered subtypes will also be detected [29,36]. Therapeutically, the mAbs reported could serve as leads for development of a neutralizing mAb combination, for example via humanization, as has been reported for BoNT/A [22]. While we did not evaluate the ability of the mAbs reported here to neutralize BoNT/F3, F5 and F6, due to lack of availability of the toxins, the mAb affinities for these subtypes would suggest that potent neutralization would be achieved. Compared to antitoxins generated by animal immunizations, such mAb combinations are more potent, can be engineered to have broader subtype coverage as reported here and are expected to have a longer half-life *in vivo* reducing the risk of reintoxication [20].

## Supporting information

S1 Fig Analysis of the purity of recombinant BoNT/F domains. **A.** Cartoon of botulinum neurotoxin primary structure. **B.** SDS-PAGE analysis of BoNT/F fragments expressed from *E*. *coli* BL21 (DE3). The upper panel shows the expression of BoNT/F LC-HN fragments fused with a 6 x His tag (~110Kd). **C.** Expression of BoNT/F HC fragments fused with a 6 x His tag (BoNT/F1 HC and BoNT/F7 HC, (~50Kd) or fused to a maltose binding protein (MBP) tag (BoNT/F2, F3, F4, F5 and F6 HC, (~80Kd).
(TIF)

S2 Fig Cross reactivity of selected mAbs against BoNT/F subtypes. Yeast displayed scFv were incubated with BoNT/F LC-H_N_ fragments (scFv 4E17, 46E12, 46.25 and 47A120) or were incubated with BoNT/F H_C_ fragments (6F8 and 28H4) and then an Alexa-647 labeled secondary IgG was used for binding detection. An Alexa-488 labeled anti-SV5 IgG was used to detect scFv mAb expression on yeasts. Binding to BoNT/F4 was measured using crude BoNT/F4 culture supernatant due to the absence of recombinant domains.
(TIF)

S3 Fig Domain specificity of cross-reactive mAbs. Yeast displayed BoNT/F1 LC, HN, LC-HN or HC was incubated with Alexa-647 labeled IgG and Alexa-488 labeled anti-SV5 IgG.
(TIF)

S1 Table Characteristics of yeast display libraries used for BoNT/F mAb generation. (PDF)

## References

[1] Control CfD (1998) Botulism in the United States, 1899–1998 handbook for epidemiologists, clinicians,and laboratory workers. Atlanta, Georgia U.S. Department of Health and Human Services, Public Health Service: downloadable at http://www.bt.cdc.gov/agent/botulism/index.asp.

[2] Gill MD (1982) Bacterial toxins: a table of lethal amounts. Microbiol Rev 46: 86–94. 6806598 10.1128/mr.46.1.86-94.1982 PMC373212

[3] Maslanka SE, Luquez C, Dykes JK, Tepp WH, Pier CL, Pellett S, et al (2015) A Novel Botulinum Neurotoxin, Previously Reported as Serotype H, Has a Hybrid-Like Structure With Regions of Similarity to the Structures of Serotypes A and F and Is Neutralized With Serotype A Antitoxin. J Infect Dis. 10.1093/infdis/jiv327 PMC4704661 26068781

[4] Lacy DB, Stevens RC (1999) Sequence homology and structural analysis of the Clostridial neurotxins. J Mol Biol 291: 1091–1104. 10.1006/jmbi.1999.2945 10518945

[5] Barash JR, Arnon SS (2014) A novel strain of Clostridium botulinum that produces type B and type H botulinum toxins. J Infect Dis 209: 183–191. 10.1093/infdis/jit449 24106296

[6] Moller V, Scheibel I (1960) Preliminary report on the isolation of an apparently new type of CI. botulinum. Acta Pathol Microbiol Scand 48: 80 14423425 10.1111/j.1699-0463.1960.tb04741.x

[7] Burke GS (1919) The Occurrence of Bacillus botulinus in Nature. J Bacteriol 4: 541–553. 16558851 10.1128/jb.4.5.541-553.1919 PMC378819

[8] Hill KK, Smith TJ (2013) Genetic diversity within Clostridium botulinum serotypes, botulinum neurotoxin gene clusters and toxin subtypes. Curr Top Microbiol Immunol 364: 1–20. 10.1007/978-3-642-33570-9_1 23239346

[9] Hill KK, Smith TJ, Helma CH, Ticknor LO, Foley BT, Svensson RT, et al (2007) Genetic diversity among Botulinum Neurotoxin-producing clostridial strains. J Bacteriol 189: 818–832. 10.1128/JB.01180-06 17114256 PMC1797315

[10] Raphael BH, Choudoir MJ, Luquez C, Fernandez R, Maslanka SE (2010) Sequence diversity of genes encoding botulinum neurotoxin type F. Appl Environ Microbiol 76: 4805–4812. 10.1128/AEM.03109-09 20511432 PMC2901728

[11] Smith TJ, Lou J, Geren IN, Forsyth CM, Tsai R, Laporte SL, et al (2005) Sequence variation within botulinum neurotoxin serotypes impacts antibody binding and neutralization. Infect Immun 73: 5450–5457. 10.1128/IAI.73.9.5450-5457.2005 16113261 PMC1231122

[12] Arnon SS, Schechter R, Inglesby TV, Henderson DA, Bartlett JG, Ascher MS, et al (2001) Botulinum toxin as a biological weapon: medical and public health management. JAMA 285: 1059–1070. 11209178 10.1001/jama.285.8.1059

[13] Franz DR, Pitt LM, Clayton MA, Hanes MA, Rose KJ (1993) Efficacy of prophylactic and therapeutic administration of antitoxin for inhalation botulism In: DasGupta BR, editor. Botulinum and Tetanus Neurotoxins: Neurotransmission and Biomedical Aspects. New York: Plenum Press pp. 473–476.

[14] Black RE, Gunn RA (1980) Hypersensitivity reactions associated with botulinal antitoxin. Am J Med 69: 567–570. 7191633 10.1016/0002-9343(80)90469-6

[15] Hibbs RG, Weber JT, Corwin A, Allos BM, Abd el Rehim MS, Sharkawy SE, et al (1996) Experience with the use of an investigational F(ab')2 heptavalent botulism immune globulin of equine origin during an outbreak of type E origin in Egypt. Clin Infect Dis 23: 337–340. 8842274 10.1093/clinids/23.2.337

[16] Arnon SS (1993) Clinical trial of human botulism immune globulin In: DasGupta BR, editor. Botulinum and Tetanus Neurotoxins: Neurotransmission and Biomedical Aspects. New York: Plenum Press pp. 477–482.

[17] Arnon SS, Schechter R, Maslanka SE, Jewell NP, Hatheway CL (2006) Human botulism immune globulin for the treatment of infant botulism. N Engl J Med 354: 462–471. 10.1056/NEJMoa051926 16452558

[18] Arnon SS (2007) Creation and development of the public service orphan drug Human Botulism Immune Globulin. Pediatrics 119: 785–789. 10.1542/peds.2006-0646 17403850

[19] (2013) Full prescribing information: Botulism Antitoxin Heptavalent (A, B, C,D, E, F, G)—(Equine).

[20] Fagan RP, Neil KP, Sasich R, Luquez C, Asaad H, Maslanka S, et al (2011) Initial recovery and rebound of type f intestinal colonization botulism after administration of investigational heptavalent botulinum antitoxin. Clin Infect Dis 53: e125–128. 10.1093/cid/cir550 21896700

[21] Nowakowski A, Wang C, Powers DB, Amersdorfer P, Smith TJ, Montgomery VA, et al (2002) Potent neutralization of botulinum neurotoxin by recombinant oligoclonal antibody. Proc Natl Acad Sci U S A 99: 11346–11350. 10.1073/pnas.172229899 12177434 PMC123259

[22] Nayak SU, Griffiss JM, McKenzie R, Fuchs EJ, Jurao RA, An AT, et al (2014) Safety and pharmacokinetics of XOMA 3AB, a novel mixture of three monoclonal antibodies against botulinum toxin A. Antimicrob Agents Chemother 58: 5047–5053. 10.1128/AAC.02830-14 24913160 PMC4135817

[23] Meng Q, Garcia-Rodriguez C, Manzanarez G, Silberg MA, Conrad F, Bettencourt J, et al (2012) Engineered domain-based assays to identify individual antibodies in oligoclonal combinations targeting the same protein. Anal Biochem 430: 141–150. 10.1016/j.ab.2012.08.005 22922799 PMC4209713

[24] Meng Q, Li M, Silberg MA, Conrad F, Bettencourt J, To R, et al (2012) Domain-based assays of individual antibody concentrations in an oligoclonal combination targeting a single protein. Anal Biochem 421: 351–361. 10.1016/j.ab.2011.09.030 22037290 PMC4209596

[25] Fan Y, Geren IN, Dong J, Lou J, Wen W, Conrad F, et al (2015) Monoclonal Antibodies Targeting the Alpha-Exosite of Botulinum Neurotoxin Serotype/A Inhibit Catalytic Activity. PLoS One 10: e0135306 10.1371/journal.pone.0135306 26275214 PMC4537209

[26] Fan Y, Dong J, Lou J, Wen W, Conrad F, Geren IN, et al (2015) Monoclonal Antibodies that Inhibit the Proteolytic Activity of Botulinum Neurotoxin Serotype/B. Toxins (Basel) 7: 3405–3423. 26343720 10.3390/toxins7093405 PMC4591640

[27] Dong J, Thompson AA, Fan Y, Lou J, Conrad F, Ho M, et al (2010) A single-domain llama antibody potently inhibits the enzymatic activity of botulinum neurotoxin by binding to the non-catalytic alpha-exosite binding region. J Mol Biol 397: 1106–1118. 10.1016/j.jmb.2010.01.070 20138889 PMC2903050

[28] Razai A, Garcia-Rodriguez C, Lou J, Geren IN, Forsyth CM, Robles Y, et al (2005) Molecular evolution of antibody affinity for sensitive detection of botulinum neurotoxin type A. J Mol Biol 351: 158–169. 10.1016/j.jmb.2005.06.003 16002090

[29] Fan Y, Barash JR, Lou J, Conrad F, Marks JD, Arnon SS (2016) Immunological Characterization and Neutralizing Ability of Monoclonal Antibodies Directed Against Botulinum Neurotoxin Type H. J Infect Dis 213: 1606–1614. 10.1093/infdis/jiv770 26936913 PMC4837907

[30] Pettersen EF, Goddard TD, Huang CC, Couch GS, Greenblatt DM, Meng EC, et al (2004) UCSF Chimera--a visualization system for exploratory research and analysis. J Comput Chem 25: 1605–1612. 10.1002/jcc.20084 15264254

[31] Corpet F (1988) Multiple sequence alignment with hierarchical clustering. Nucleic Acids Res 16: 10881–10890. 2849754 10.1093/nar/16.22.10881 PMC338945

[32] Robert X, Gouet P (2014) Deciphering key features in protein structures with the new ENDscript server. Nucleic Acids Res 42: W320–324. 10.1093/nar/gku316 24753421 PMC4086106

[33] Garcia-Rodriguez C, Geren IN, Lou J, Conrad F, Forsyth C, Wen W, et al (2011) Neutralizing human monoclonal antibodies binding multiple serotypes of botulinum neurotoxin. Protein Eng Des Sel 24: 321–331. 10.1093/protein/gzq111 21149386 PMC3038462

[34] Garcia-Rodriguez C, Levy R, Arndt JW, Forsyth CM, Razai A, Lou J, et al (2007) Molecular evolution of antibody cross-reactivity for two subtypes of type A botulinum neurotoxin. Nat Biotechnol 25: 107–116. 10.1038/nbt1269 17173035

[35] Grossman I, Ilani T, Fleishman SJ, Fass D (2016) Overcoming a species-specificity barrier in development of an inhibitory antibody targeting a modulator of tumor stroma. Protein Eng Des Sel 29: 135–147. 10.1093/protein/gzv067 26819240 PMC4795942

[36] Kalb SR, Garcia-Rodriguez C, Lou J, Baudys J, Smith TJ, Marks JD, et al (2010) Extraction of BoNT/A, /B, /E, and /F with a single, high affinity monoclonal antibody for detection of botulinum neurotoxin by Endopep-MS. PLoS One 5: e12237 10.1371/journal.pone.0012237 20808925 PMC2923190

