# Supplementary figures and images for: A three monoclonal antibody combination potently neutralizes multiple botulinum neurotoxin serotype F subtypes
Source: PLoS One. 2017 Mar 21;12(3):e0174187. doi: 10.1371/journal.pone.0174187 (PMC5360321; doi:10.1371/journal.pone.0174187)

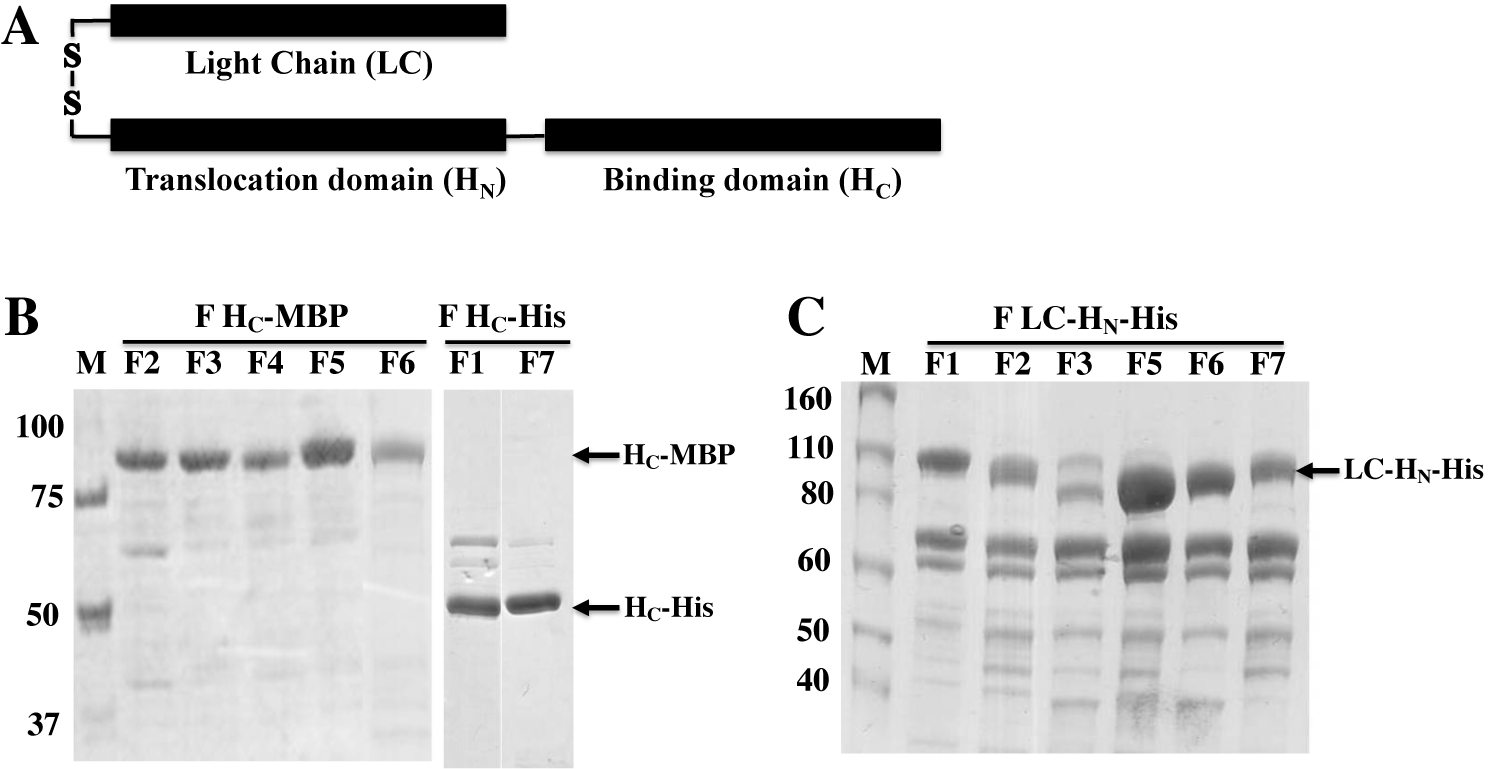

Supplement: S1 Fig — A. Cartoon of botulinum neurotoxin primary structure. B. SDS-PAGE analysis of BoNT/F fragments expressed from E. coli BL21 (DE3). The upper panel shows the expression of BoNT/F LC-HN fragments fused with a 6 x His tag (~110Kd). C. Expression of BoNT/F HC fragments fused with a 6 x His tag (BoNT/F1 HC and BoNT/F7 HC, (~50Kd) or fused to a maltose binding protein (MBP) tag (BoNT/F2, F3, F4, F5 and F6 HC, (~80Kd). (TIF) [file pone.0174187.s001.tif]

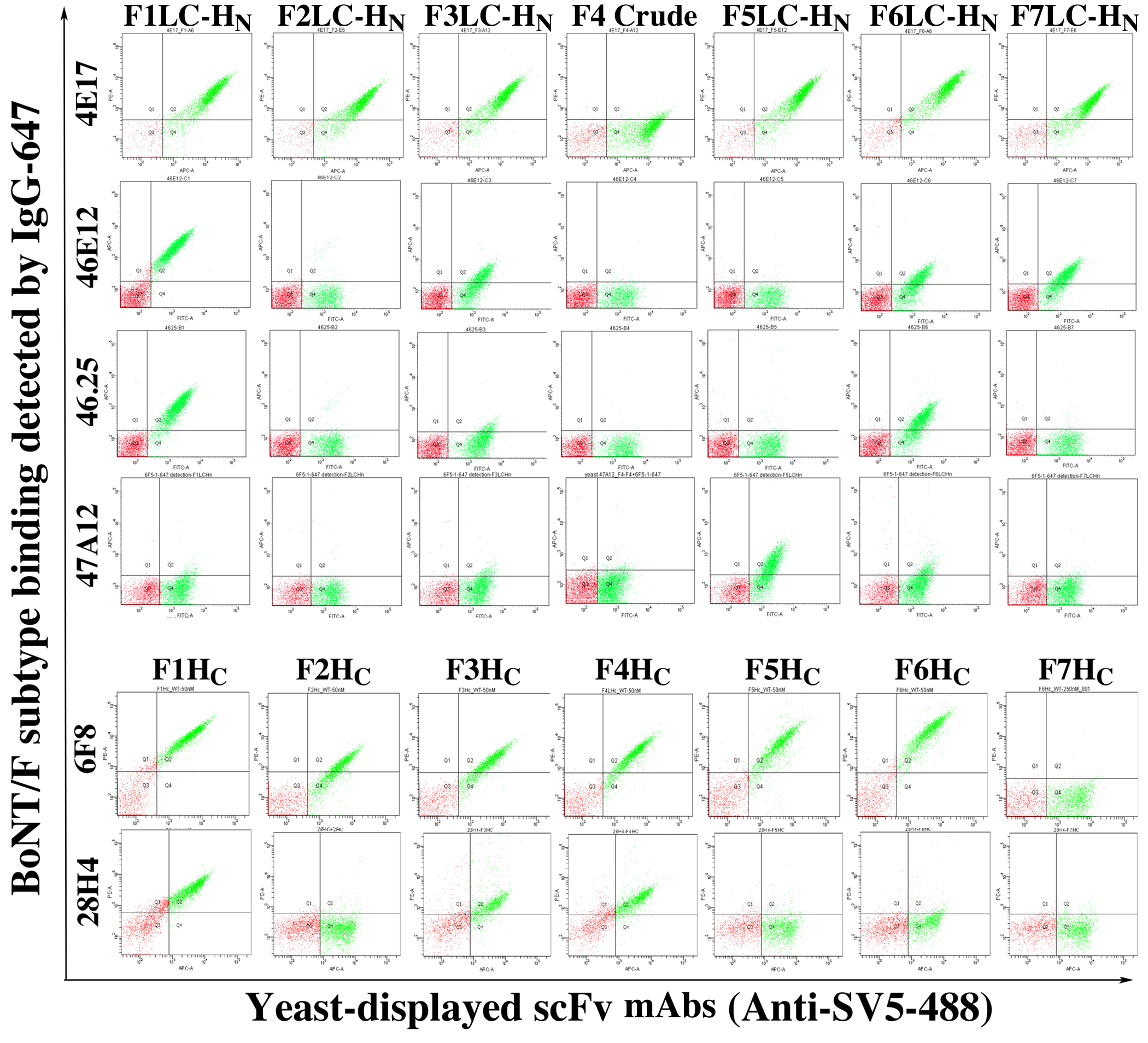

Supplement: S2 Fig — Yeast displayed scFv were incubated with BoNT/F LC-HN fragments (scFv 4E17, 46E12, 46.25 and 47A120) or were incubated with BoNT/F HC fragments (6F8 and 28H4) and then an Alexa-647 labeled secondary IgG was used for binding detection. An Alexa-488 labeled anti-SV5 IgG was used to detect scFv mAb expression on yeasts. Binding to BoNT/F4 was measured using crude BoNT/F4 culture supernatant due to the absence of recombinant domains. (TIF) [file pone.0174187.s002.tif]

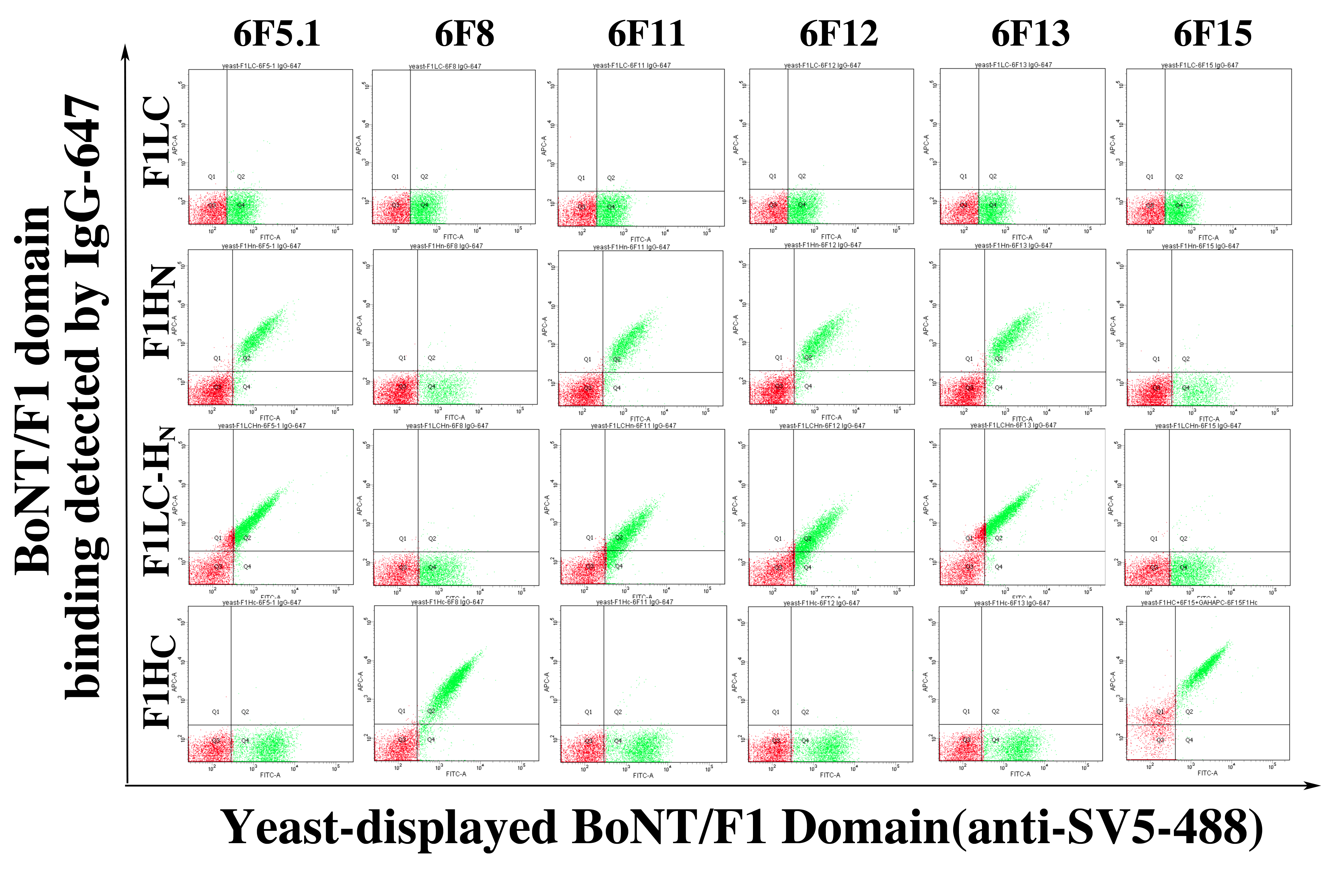

Supplement: S3 Fig — Yeast displayed BoNT/F1 LC, HN, LC-HN or HC was incubated with Alexa-647 labeled IgG and Alexa-488 labeled anti-SV5 IgG. (TIF) [file pone.0174187.s003.tif]
