# Supplementary material for: A three monoclonal antibody combination potently neutralizes multiple botulinum neurotoxin serotype F subtypes
Source: PLoS One. 2017 Mar 21;12(3):e0174187. doi: 10.1371/journal.pone.0174187 (PMC5360321; doi:10.1371/journal.pone.0174187)
Supplement: S1 Table — (PDF) [file pone.0174187.s004.pdf]

Supplementary Table 1. Characteristics of yeast display libraries used for BoNT/F mAb generation.

Library name	Initial immunogen	Boosting immunogen	Library size
Mouse BoNT/F1H_C	BoNT/F1 H_C	BoNT/F1 H_C	$7.6x10^7$
Mouse BoNT/F1	BoNT/F1 H_C	BoNT/F1 toxin complex	$9.0x10^7$
Mouse BoNT/F3 LC-H_N	BoNT/F3 LC-H_N	BoNT/F3 LC-H_N	$1.8x10^7$
Mouse BoNT/F5 LC-H_N	BoNT/F5 LC-H_N	BoNT/F5 LC-H_N	$2.0x10^7$
Mouse BoNT/F6 LC-N_N	BoNT/F6 LC-H_N	BoNT/F6 LC-H_N	$1.6x10^7$
Mouse BoNT/F7 H_C	BoNT/F7 H_C	BoNT/F7 H_C	$3x10^7$
Mouse BoNT/F7Lc-H_N	BoNT/F7 LC-H_N	BoNT/F7 LC-H_N	$4x10^7$
Human Donors 28, 29, 30 Mixture	Pentavalent BoNT Toxoid	Pentavalent BoNT Toxoid	$2x10^{8}$
